# Infection control and risk factors for acquisition of carbapenemase-producing enterobacteriaceae. A 5 year (2011–2016) case-control study

**DOI:** 10.1186/s13756-019-0668-2

**Published:** 2020-01-17

**Authors:** Luigi Segagni Lusignani, Elisabeth Presterl, Beata Zatorska, Miriam Van den Nest, Magda Diab-Elschahawi

**Affiliations:** Department of Infection Control and Hospital Epidemiology, Medical University of Vienna, Waehringer Guertel 18-20, 1090 Vienna, Austria

**Keywords:** Enterobacteriaceae, Carbapenemase, Epidemiology, Screening, Infection control

## Abstract

**Background:**

Carbapenemase-producing enterobacteriaceae (CPE) are a major threat for severely ill patients. However, only limited data on the epidemiology and on evidence-based infection prevention and control measures are available. The aim of this study was to investigate the epidemiology of patients with CPE, characterizing the CPE isolates by their resistance mechanisms and genetic similarity, to explore risk factors for their acquisition, and to evaluate the effectiveness of the current CPE infection control measures.

**Methods:**

A retrospective case-control study was performed using data from 2011 to 2016 in a 1800-bed academic hospital in Central Europe, where risk-based screening at patients´ admission is performed. Carbapenem resistance mechanisms of all carbapenem resistant enterobacteriaceae from patients admitted during this period were investigated. Clinical data of the CPE-positive patients were analysed and compared to a matched control group (case-control ratio of 1:3). We performed univariate and multivariate statistical analysis to identify risk factors for CPE acquisition.

**Results:**

Of 621,623 admitted patients in the study period, 75 patients with carriage of carbapenem resistant enterobacteriaceae were included (0.12/1000 admittances). Carbapenemase-encoding genes were detected in 77.3% (58/75) of patients with carbapenem-resistant enterobacteriaceae. The enzyme *bla*OXA-48 was found in 34.5% (20/58), *bla*KPC in 29.3% (17/58), *bla*NDM enzymes in 20.7% (12/58) and *bla*VIM in 8.6% (5/58) of the isolates. The overall mortality among CPE patients was 25.9% (15/58) and attributable mortality of CPE was 53.3% (8/15). Multivariate analysis revealed four risk factors to be independent predictors of CPE carriage: the length of hospital admission > 20 days (AOR: 4.9, 95% CI: 1.4–15.5; *P* <  0.001), hospital admission within the previous year (AOR: 22.3, 95% CI: 3.9–88.4; P <  0.001), exposure to a healthcare facility in a country with high or unknown carbapenem-resistant enterobacteriaceae prevalence 3 months before admission (AOR: 11.8, 95% CI: 2.2–63.2; *P* <  0.01) and the use of antibiotics longer than 10 days (AOR: 5.2, 95% CI: 1.4–35.9; *P* <  0.05). The current risk-based screening strategy at hospital admission could not identify 37 (63.8%) of the 58 CPE-positive patients. Epidemiological investigation and genotyping revealed that no outbreaks due to CPE occurred during this period.

**Conclusion:**

Overall, the CPE carriage rate in patients was very low, the attributable mortality, however, is alarming (53%). *Bla*OXA-48 and *bla*KPC were the main cause of carbapenem resistance in enterobacteriaceae. Although the strict application of standard infection control measures was effective for prevention of outbreaks in this setting, an enlarged risk based targeted screening strategy has to be implemented.

## Introduction

Carbapenem-resistant *enterobacteriaceae* have recently been listed by the World Health Organisation (WHO) as antibiotic-resistant priority pathogens [1]. Infections with carbapenem-resistant enterobacteriaceae are difficult to treat and may cause death in up to 50% of infected patients [2]. The major mechanism for carbapenem resistance in enterobacteriaceae is the production of enzymes of the class carbapenemases. Carbapenemases are β–lactamase enzymes with the ability to hydrolyse most β-lactam-antibiotics, including carbapenems. Carbapenemases are classified into Ambler class A, class B and class D [3]. Among them, the most broadly spread carbapenemases are KPC, VIM, IMP, NDM and OXA-48 [4]. Frequently, carbapenemase-producing enterobacteriaceae are multidrug-resistant, because they also harbour genes encoding for resistance against other antibiotic classes, such as fluoroquinolones and aminoglycosides [2].

CPE are spreading globally, and their prevalence seems to vary widely between continents and countries [5, 6]. Based on the European Antimicrobial Resistance Surveillance Network (EARS-Net) protocols, national surveillance systems of CPE have been implemented since 2010 in many European member states [7], and CPE prevalence was found to be especially high in South-Eastern Europe, possibly due to a delay in strict monitoring [8].

Perception of risk factors for CPE is important to install a targeted screening strategy of patients at risk, to implement appropriate infection control measures and, if necessary, empiric antimicrobial therapy. To date, prior antimicrobial exposure, the length of hospital stay, the presence of invasive devices, advanced age and severe underlying diseases were identified as independent risk factors for CPE carriage [9–19]. Recent studies have focused on investigating CPE colonization of patients in cross-border transfers, but these results are debatable due to paucity of evidence [17, 20–23]. Moreover, since a risk-based strategy of targeted screening relies heavily on accurate history taking, full disclosure from the patient and mutual understanding, it remains a challenge in the clinical routine [24].

The aims of this study were to investigate the presence of carbapenemases in all carbapenem-resistant enterobacteriaceae isolated in the years 2011–2016, to identify carbapenemases using molecular methods, to identify risk factors for the presence of CPE and to evaluate if current infection control measures are effective in both detection and containment of CPE.

## Methods

### Setting

This study was conducted at the academic teaching hospital of the Medical University of Vienna (VGH). It harbours Europe’s largest lung transplant facility and a large hemato-oncological unit. The hospital provides 1782 beds, including 130 intensive-care unit beds and 137 intermediate-care beds. Although the main part of the patients cared for are Austrians, there is a considerable amount of patients transferred from South and Eastern Europe, Mediterranean countries and East Asia. Between 2011 and 2016, the number of admitted patients increased by 14.4%, reaching 114,029 admissions in 2016.

### Characterization of the bacterial isolates

**Fig. 1:**
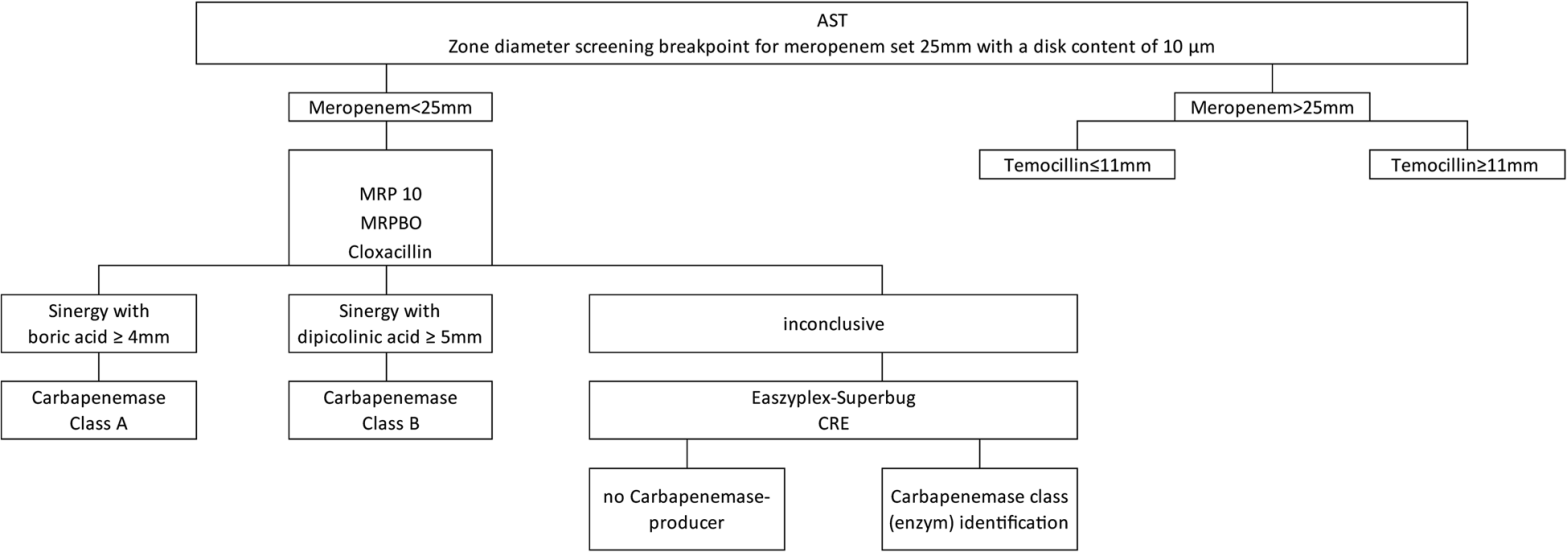
Algorithm used for detection of carbapenemases in enterobacteriaceae. Legend: The temocillin test is only valid for enterobacteriaceae; if both meropenem and all combinations show no zone of inhibition, the temocillin test is invalid, and the result inconclusive

The initial carbapenem-resistant enterobacteriaceae isolates of patients admitted between 1st July 2011 and 30th June 2016 in VGH were included in the study. The bacterial isolates were cultivated on Columbia agar with 5% sheep blood (bioMérieux, L’étoile, France). Antimicrobial susceptibility testing was performed according to the protocols of EUCAST (http://www.eucast.org). To distinguish between carbapenemase and ESBL producers, we performed a screening assay comprising chromID ESBL (bioMérieux Germany) and chromID CARBA SMART (bioMérieux Germany) agar plates. The Rosco kit (ROSCO Diagnostica A / S, Denmark) was used to screen for the presence of a carbapenemase. The eazyplex® SuperBug kit (Amplex Diagnostics GmbH, Germany) was used to genetically identify the carbapenemases and to categorize them into the carbapenemase classes (Fig. 1).

### Case-control study to identify risk factors

Epidemiological, demographic and clinical data of patients with CPE were collected from the patients’ electronic medical records, recorded in an Infection Prevention and Control (IPC) devoted database and analysed. A retrospective case-control study with a case-control ratio of 1:3 was performed. Controls were matched to the cases according to: a) gender, b) age plus/minus one month, and c) the hospital admission date plus/minus one day. If multiple control patients fitted the inclusion criteria, the one who had been admitted closest to the case patient, was retained. Controls, which had a previous history of carbapenem-resistant enterobacteriaceae, were excluded from the recruitment. A total of 177 patients served as controls. The following variables identified in literature as risk factors for CPE acquisition [9–19] were collected:

- Ward of hospital stay;
- History of hospitalization;
- Length of stay;
- Surgical interventions;
- Exposure to invasive devices;
- Presence of comorbidity;
- Exposure to antimicrobial agents;
- Contact with healthcare system of countries with high or unknown carbapenem-resistant enterobacteriaceae prevalence.

Hospital wards were categorised into medical, surgical and “high-risk” wards. High-risk wards included adult intensive-care units, the neonatology units, the transplant and the burns intensive-care units. Patients’ hospitalization history comprised the evaluation of the patients´ admission origin (from home, from a national or foreign hospital) and any admission to a hospital within the previous year. The length of stay was defined as the period from admission to the VGH until the date of the first isolation of CPE for cases, and from admittance until discharge for controls, in days. Invasive devices like mechanical ventilation and central vascular catheters were recorded. Data on invasive devices and surgical interventions, as well as data on exposure to carbapenems and length of exposure of any antimicrobial agents, were collected for a period starting three months before VGH hospitalization.

The presence of comorbidity was evaluated using Charlson Comorbidity Index (CCI) score.

Previous contact with foreign healthcare systems included any kind of admittance, outpatient consultation, treatment or invasive procedure in the last three months before admission to the VGH, independent of the kind of healthcare setting and the time spent there. High-prevalence countries for carbapenem-resistant enterobacteriaceae were defined according to EARS-Net reports from 2011 to 2015 [8]. Patients’ mortality was assessed for the period of admittance to the VGH only. Mortality was considered attributable to CPE infection if the patient died within one week of CPE infection onset and: a) patient’s death was due to a septic shock, or b) CPE infection as cause of death was clearly stated in the medical record.

### Infection control practice and outbreak investigation

To identify carriers of CPE and to implement infection control measures to contain the spread of CPE, the VGH has adopted the policy of a risk based targeted screening for CPE since 2011. The targeted rectal screening has to be performed in admitted patients who have at least one the following risk factors: 1) contact with the healthcare system of foreign countries or a stay in foreign countries prior to admittance, particularly in carbapenem-resistant enterobacteriaceae high-prevalence countries, 2) previous treatment with carbapenems, 3) previous history of carbapenem-resistant enterobacteriaceae colonization. At the VGH, stringent standard IPC measures are generally executed. If a patient with CPE is identified, more elaborate IPC measures have to be performed according to the hospital wide CPE guideline. Every case of CPE is immediately reported to the infection control team via the institute of microbiology. Upon notice, an IPC nurse counsels the respective ward to implement IPC measures and handles additional questions of the health care workers on CPE, then monitors and records all implemented IPC measures in the IPC database (Research Data Analysis Software, IT4Science, Medical University of Vienna). Besides standard hygiene measures, every CPE-positive patient is isolated in a single-bed room with an allocated individual toilet. In this designated room, personnel and visitors are obliged to wear personal protective equipment including long-sleeved protective gown, gloves, mask and cap. In addition, both, CPE patients and visitors, are counselled on CPE and appropriate infection control measures by the infection control nurse.

Routinely, upon the occurrence of CPE in clinical specimens, an epidemiological outbreak investigation and screening of the contact patients were performed.

For the present analysis, a retrospective epidemiological investigation was conducted, including the construction of an epidemic curve to display incident cases and possible transmissions. A transmission of CPE was considered possible if CPE-positive patients were admitted to the same ward and during the same period of time. In order to determine genetic relatedness of the CPE strains, molecular genotyping was performed by means of an automated repetitive-sequence-based PCR (rep-PCR) assay on the DIVERSILAB®system (DL, bioMérieux, France). A cut-off at 95% of the Dice coefficient of similarity by rep-PCR was used to identify identical strains and to verify CPE transmission.

### Statistical analysis

Descriptive analyses were performed to summarize both patients and specimen information. Chi-Square -Test (χ2) and exact Fisher’s test were used to compare proportions when applicable. A logistic regression model was used to compare CPE and non-CPE patients. All significant variables identified in the univariate analysis were included in a stepwise selection multivariate logistic regression model, to adjust the confounding factors if *P* <  0.001. Data analysis was performed using SPSS Statistics 23.0 (IBM Corp, Armonk, NY, USA). A *P* value < 0.05 was considered significant.

## Results

### Carbapenem resistance and characterization of carbapenemases

**Fig. 2:**
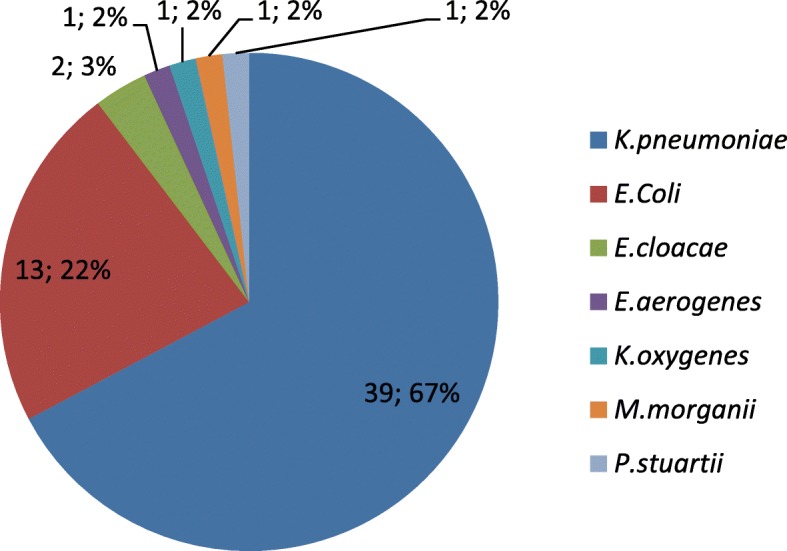
Carbapenemase-producing enterobacteriaceae. Legend: Number and percentage of isolated CPE

The presence of carbapenemases was identified in 77.3% (58/75) of carbapenem-resistant enterobacteriaceae. The number of CPE isolates increased from 3 per 50,000 hospital admissions in the last six months of 2011 to 24 per 50,000 hospital admissions in the first six month of 2016. The bacterial species are shown in Fig. 2. Seventeen (22.6%) isolates additionally produced extended-spectrum β-lactamases (ESBL). The Amber classification of carbapenemase enzymes is shown in Table 1.

**Table 1:** Distribution of carbapenemase Amber classes and carbapenemase enzymes

Amber class	Enzyme	No.	Percentage (%)
A	KPC	12	20.7
	KPC + CTX-M1	3	5.2
	KPC + AmpC	2	3.4
B	NDM	5	8.6
	NDM + CTX-M1	6	10.3
	NDM + AmpC	1	1.7
	VIM	3	5.2
	VIM + CTX-M1	1	1.7
	VIM + CTX-M1 + AmpC	1	1.7
D	OXA-48	6	10.3
	OXA-48 + CTX-M1	8	13.8
	OXA-48 + CTX-M9	1	1.7
	OXA-48 + AmpC	2	3.4
	OXA-48 + CTX-M1 + AmpC	3	5.2
B + D	NDM + OXA-48	2	3.4
	NDM + OXA-48 + CTX-M1	1	1.7
	NDM + OXA-48 + CTXM9	1	1.7
Total		58	100.0

### CPE patients analysis - clinical and epidemiological data

**Fig. 3:**
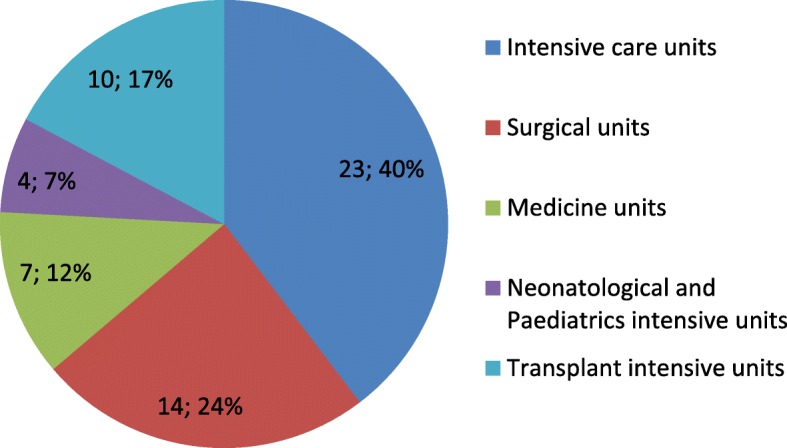
Carbapenemase-producing enterobacteriaceae ward of isolation. Legend: Number of isolated CPE and their relative frequencies (percentage)

**Fig. 4:**
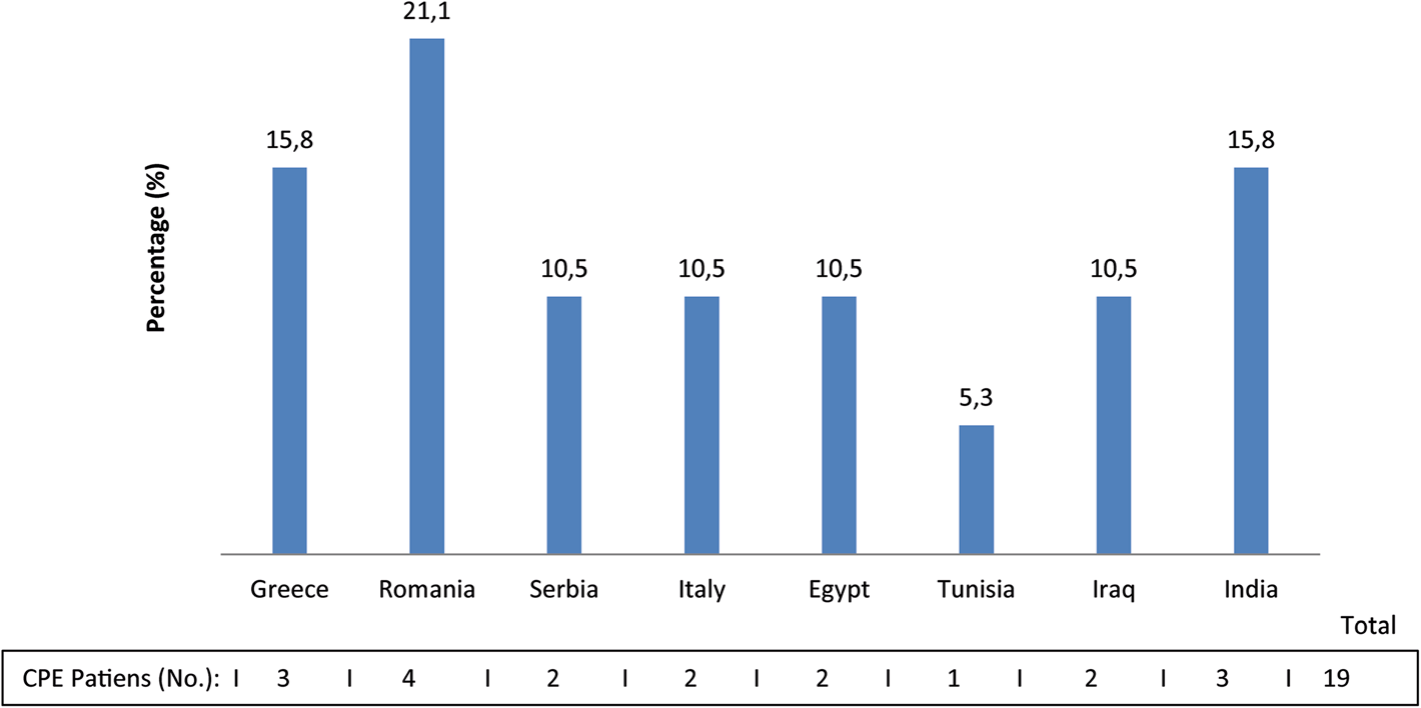
Distribution of CPE positive patients by contact with foreign healthcare system. Legend: Number and percentage of the 19 CPE patients who had previous contact with a healthcare system of foreign countries

Median age of the 58 CPE patients was 48 years (IQR: 32–61) and 44.8% (26/58 of cases and 79/177 of controls) were females. The wards where CPE were isolated are shown in Fig. 3. The CPE patients had a median length of stay in the ICUs of 77 days (IQR: 38–91). Figure 4 shows the countries where CPE positive patients had had contact with the healthcare system.

Out of the 58 patients, 47% (27/58) were initially only colonized with CPE and 53.4% (31/58) had an infection caused by CPE. Consequently, nineteen of the 27 CPE patients (70.4%) who were colonized developed an infection during their hospitalization period.

The median time between the first isolation of CPE and death or discharge was 29 days (IQR 95%: 14–58). The crude mortality in CPE patients (15/58; 25.9%) was significantly higher than that in the control group (14/177; 7.9%) (χ2 exact Fisher’s test: 13.0; *P* <  0.001). The attributable mortality associated with CPE infections was 53.3% (8/15). Of the eight patients who died because of infection with CPE, five (62%) were previously colonized by CPE.

### Antimicrobial exposure

Thirty-one of the 58 CPE-positive patients (53.4%) had received antimicrobial therapy within the three months before isolation of CPE. Overall, 42 antimicrobials were administrated (Additional file 1) and eighteen patients were treated with two or more antimicrobials. The median duration of antimicrobial therapy before isolation of CPE was 20 days (IQR 95%: 7–25). Previous carbapenem exposure and length of exposure to antibiotics are described in Table 2.

**Table 2:** Univariate analysis of factors influencing the acquisition of carbapenemase-producing enterobacteriaceae

Variables		CPE (%)	Controls (%)	OR^a^	*P* value
Ward of hospital stay	Medicine	7 (6.5%)	100 (93.5%)	Ref.^b^	
	High-risk	37 (77.1%)	11 (22.9%)	48.0 (17.3–133.2)	< 0.001
	Surgery	14 (17.5%)	66 (82.5%)	15.8 (6.5–38.4)	< 0.05
LOS^c^	< 20 days	31 (16.9%)	152 (83.1%)	Ref.^b^	
	≥ 20 days	27 (51.9%)	25 (48.1%)	5.3 (2.7–10.3)	< 0.001
Admission origin	Home	36 (18.8%)	155 (81.2%)	Ref.^b^	
	Non-Austrian hospital	12 (85.7%)	2 (14.3%)	25.8 (5.5–120.5)	< 0.001
	Austrian hospital	10 (33.3%)	20 (66.7%)	12 (2.2–64.3)	ns
Hospital admission in the previous year^d^	none	18 (10.8%)	148 (89.2%)	Ref.^b^	
	yes	40 (58.0%)	29 (42.0%)	11.4 (5.7–22.4)	< 0.001
Surgical interventions	none	49 (22.5%)	169 (77.5%)	Ref.^b^	ns
	yes	9 (52.9%)	8 (47.1%)	3.9 (1.4–10.6)	< 0.001
Mechanical ventilation (MV)	none	33 (17.6%)	154 (82.4%)	Ref.^b^	
	yes	25 (52.1%)	23 (47.9%)	5.1 (2.6–10.0)	< 0.001
Central vascular catheter (CVC)	none	19 (12.7%)	131 (87.3%)		
	yes	39 (45.9%)	46 (54.1%)	5.8 (3.1–11.1)	< 0.001
Presence of co-morbidity (CCI^e^)	< 4	43 (22.5%)	148 (77.5%)		
	≥4	15 (34.1%)	29 (65.9%)	1.8 (0.8–3.6)	ns
Carbapenem exposure^f^	none	46 (79.3%)	174 (98.3%)	Ref. ^b^	
	yes	12 (20.7%)	3 (1.7%)	15.1 (4.1–55.9)	< 0.001
Antimicrobial therapy > 10 days^f^	none	27 (46.6%)	144 (29.3%)	Ref. ^b^	
	> 10 days	14 (24.1%)	4 (2.3%)	18.6 (5.7–61.0)	< 0.001
	< 10 days	17 (29.3%)	29 (16.4%)	5.9 (1.7–21.1)	ns
Contact with healthcare system of high-risk country^f,g^	none	39 (19.0%)	166 (81.0%)	Ref. ^b^	
	yes	19 (63.3%)	11 (36.7%)	6.8 (2.9–15-5)	< 0.001

^a^OR: Odds ratio, ^b^Ref.: Reference category, ^c^LOS: Length of stay, ^d^ in any hospital, ^e^CCI: Charlson comorbidity index, ^f^ 90 days before VGH admission, ^g^ Healthcare system of countries of high or unknown carbapenem-resistant enterobacteriaceae prevalence.

### Risk factors analysis

Most of the risk factors tested in the univariate analysis shown in Table 2 were associated with CPE acquisition.

Multivariate analysis of the ten selected variables identified four conditions for a higher risk of CPE acquisition (Table 3).

**Table 3:** Multivariate analysis of factors influencing the acquisition of carbapenemase-producing enterobacteriaceae

*Variable*	AOR^a^	95% IC	P
LOS^b^ ≥ 20 days	4.9	1.4–15.5.1	< 0.01
Hospital stay in the previous year^c^	22.3	3.9–88.4	< 0.001
Contact with healthcare system of high-risk country^d^	11.8	2.2–63.2	< 0.01
Antimicrobial therapy ≥10 days^d,e^	5.2	1.4–35.9	< 0.05

^a^AOR: Adjusted Odds ratio; ^b^LOS: Length of stay; ^c^in any hospital, ^d^in the previous 3 months; ^e^Healthcare system of countries of high or unknown carbapenem-resistant enterobacteriaceae prevalence.

In 21 of the 58 CPE-positive patients (36.2%), CPE were detected by VGH admission screening. Of those patients, 7 (33.3%) were transferred to the VGH from a non-Austrian hospital and 4 (19.0%) from an Austrian hospital. All CPE patients who had contact with healthcare systems of carbapenem-resistant enterobacteriaceae high-risk countries within the 90 days before VGH admission belonged to this group. In 37 of the 58 CPE-positive patients (63.8%), CPE were detected after more than 2 days (median: 28 days; IQR: 11–39; min: 4; max: 134) of stay at the VGH.

**Fig. 5:**
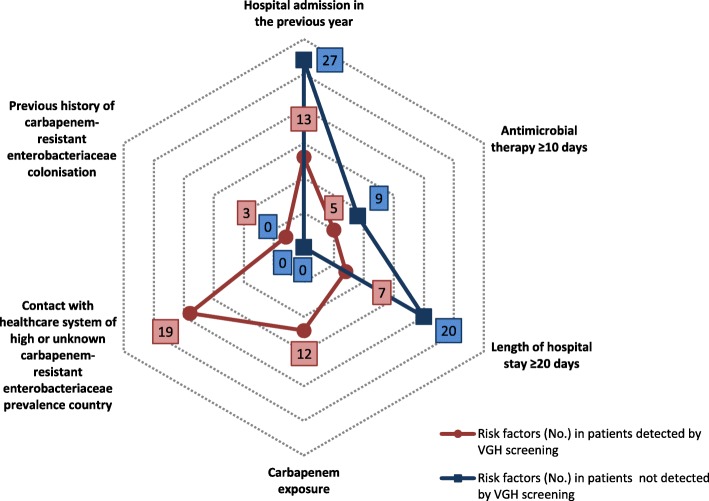
Distribution of the risk factors in CPE patients and CPE admission screening results. Legend: The spider web diagram shows the risk factors according to the targeted screening strategy plus those identified by the multivariate analysis. The asterisk indicates additional risk factors identified by the multivariate analysis

The distribution of the risk factors in the CPE patients detected at admission screening and in the ones not detected is shown in Fig. 5. Only 5.2% (3/58) of the patients had none of the risk-factors identified by the multivariate analysis.

### Outbreak investigation and infection control measures

Of the 37 patients, who were not detected by screening on admission, epidemiological analysis and investigation, as well as screening of possible contact patients were performed. A possible transmission of CPE between sixteen (43.2%) of these patients was further investigated, as they were hospitalized in the same wards during the same period of time (Additional file 2). These 16 patients had a median length of stay until CPE isolation of 26 days (IQR 95%: 9.5–41.5). In three of them, CPE were confirmed to have the same carbapenemase resistance mechanism (*Klebsiella pneumoniae bla*OXA-48). The first patient with *K.pneumoniae* OXA-48 stayed in the ward for nine weeks together with the second patient, and never with the third patient; the second patient stayed in the ward for one week together with the third patient. None of them shared the same room during their stay at the ICU. For those three patients, genotyping identified 3 distinct genotypes with a Dice Similarity Coefficient between 67.6 and 90.2%. The isolates of the 13 other patients were not genotyped, as CPE varied in resistance mechanisms. Furthermore, after screening for contact patients, no other cases were detected.

## Discussion

In this study, the most common carbapenemase enzyme among the investigated enterobacteriaceae was *bla*OXA-48, followed by *bla*KPC and *bla*NDM enzymes. Most patients with CPE were admitted to either ICUs or to, the beforehand defined, high-risk wards. This finding suggests that these wards are at risk for spread of CPE into the environment and a hotspot for transmission, if infection control precautions are not stringent and meticulously implemented.

Previous studies on CPE patients and mortality provided heterogeneous data [9, 12, 25]. Further, data on attributable mortality to CPE carriage and infection has not been reported yet. In our study, the CPE patients’ mortality was significantly higher than that of the control group patients and the attributable mortality rate of CPE infection was 53.3% (8/15). Considering the high rates of infections in previously colonized patients (70%), CPE colonization and resulting infection seem to have a high impact on mortality of patients. Thus, early detection of CPE by screening and preemptive containment of patients at risk for carrying CPE are crucial, not only to prevent outbreaks, but also to implement early adequate antimicrobial treatment [26–28].

Identification of risk factors associated with CPE colonization or infection is therefore pivotal where targeted screening is recommended [4, 29]. To date, most studies have focused on risk factors only for infections by CPE, but not for acquisition of CPE. In the present study, the multivariate analysis revealed that the increased risk of carriage of CPE was associated with 4 risk factors only. In contrast to previous studies, the presence of invasive devices was not associated with the presence of CPE [9, 17–19]. A long hospital stay was already reported to be a risk factor for CPE acquisition in other studies [11, 30]. Even though the previous length of stay in hospitals other than VGH was not investigated, almost half of the patients (41.3%) were admitted at ICUs for long periods, thus reflecting both, their critical conditions and need of life supporting procedures. In our analysis, previous hospital admissions within 365 days before detection of CPE, independently of the hospital setting was a further risk factor for CPE acquisition. Although this association has already been found in the past [16], we believe that this finding needs to be further investigated, in order to discriminate between transfers from primary, secondary, tertiary, and specialised hospitals. The referral and tertiary healthcare centres for instance are expected to have more patients carrying multi-resistant gram-negative bacteria also in countries where the CPE rates are low. Patients transferred from these hospitals could therefore be at risk for CPE acquisition and should potentially be screened.

Another risk factor for CPE acquisition was previous contact with a foreign healthcare setting with either high or unknown endemic carbapenem-resistant enterobacteriaceae situation. The first reported patients with CPE infections in the European region were indeed imported from countries with high prevalence of carbapenem-resistant enterobacteriaceae [7]. Despite limited evidence but based on the experience of controlling MRSA, the ECDC expert recommendations advise active rectal screening of patients who are transferred from a healthcare facility of another countries [7, 10]. Likewise, the results of the present study clearly support the necessity of identifying these patients in order to implement active screening and effective infection control measures, if needed. The duration of previous antimicrobial therapy longer than 10 days was a further independent risk factor for CPE carriage. The positive correlation between antimicrobial use and carbapenem resistance in enterobacteriaceae has already been described [28–31]: exposure to broad-spectrum antimicrobials including fluoquinolones, 3rd and 4th generation cephalosporins and carbapenems lead to the emergence of CPE [13–15, 31]. Therefore, continuous surveillance of antimicrobial consumption and prudent use of antimicrobials may provide a key warning indicator to anticipate increased incidence of carbapenem-resistant microorganisms [32]. Moreover, the findings of our study provide additional evidence that indicates the importance of installing antimicrobial stewardship programs [33].

Another aim of our study was to analyse the infection control measures regarding CPE at our hospital. The VGH targeted screening at admission did not detect 64% of the patients later identified as CPE positive during hospitalization. Even though universal screening for CPE may be easier to implement, it is costly and a considerable work load for the microbiological laboratory, particularly in low prevalence countries like Austria [34, 35]. In order to improve the screening at admission to the VGH in the future, the 4 risk factors identified in the multivariate analysis, will be incorporated into the targeted screening strategy.

During the whole study period, nevertheless, we detected neither CPE in-hospital transmission nor outbreaks caused by CPE at the VGH. We believe that this finding highlights the importance to implement good standard hygiene processes and rigorous infection control measures. However, further controlled studies should be conducted to evaluate the direct effects of those measures.

The strength of the present study is that patients with CPE carriage have been reviewed over a five year period and not only during single outbreaks, and, further, have been compared to a time, gender and age-matched control cohort. The principal limitation of the current study is the retrospective design. Due to the control group matching criteria, the difference in age between CPE patients and the control group was not investigated, even though higher age had been identified as a risk factor for CPE carriage in other studies [11, 36].

## Conclusion

Our study added important findings to the distribution of CPE in a tertiary care hospital receiving patients from a large part of Europe, and provided a first insight in the epidemiology of CPE carriage in this area. Patients with long and frequent hospital admissions, with previous contact with health facilities in countries with high carbapenem-resistant enterobacteriaceae prevalence or of unclear epidemiological data, and patients receiving antimicrobial treatment for ten days or longer, are more likely to acquire colonization or infection with CPE. A checklist of the identified risk factors at hospital admission will enhance the identification of patients, who should be screened. Our results also suggest that a general strict implementation of standard infection control measures, are effective in preventing the spread of CPE.

## Supplementary information

**Additional file 1.** Distribution of antimicrobials administrated to the CPE patients.

**Additional file 2.** Epidemic curve of the 16 CPE patients who were admitted in the same VGH wards at the same period of time.

## Data Availability

Not applicable.

## Abbreviations

AOR: Adjusted Odds Ratio
CCI: Charlson comorbidy index
CPE: Carbapenemase-producing enterobacteriaceae
EARS: European Antimicrobial Resistance Surveillance
ECDC: European Centre for Disease Prevention and Control
ESBL: Extended-spectrum enterobacteriaceae ß-lactamase
EUCAST: European Committee on Antimicrobial Susceptibility Testing
EuSCAPE: European Survey on Carbapenemase-producing enterobacteriaceae
ICU: Intensive care unit
IQR: Interquantile range
KPC: *Klebsiella pneumoniae* carbapenemase
LOS: Length of stay
MBL: Metallo-β-lactamases
NPWT: Negative-pressure wound therapy
OR: Odds Ratio
OXA: Oxacillinase
VGH: Vienna General Hospital
VIM: Verona integrin-encoded metallo-β-lactamases
WHO: World Health Organization
